# Association of Allergic Diseases and Related Conditions with Dietary Fiber Intake in Korean Adults

**DOI:** 10.3390/ijerph18062889

**Published:** 2021-03-12

**Authors:** Hoyoung Lee, Kijeong Lee, Serhim Son, Young-Chan Kim, Ji Won Kwak, Hyeon Geun Kim, Sang Hag Lee, Tae Hoon Kim

**Affiliations:** 1 Department of Otorhinolaryngology-Head and Neck Surgery, College of Medicine, Korea University, Seoul 02841, Korea; hyu415@naver.com (H.L.); peppermint_1111@hotmail.com (K.L.); goldhand11@nate.com (Y.-C.K.); jwon1111@naver.com (J.W.K.); gusrms20@naver.com (H.G.K.); sanghag@kumc.or.kr (S.H.L.); 2 Department of Biostatistics, Korea University College of Medicine, Seoul 02841, Korea; sonserhim@korea.ac.kr

**Keywords:** dietary fiber, allergic disease, nutrition survey, asthma, allergic rhinitis, atopic dermatitis, adult

## Abstract

An association between fiber intake and allergic diseases in children has been reported; however, many studies have not been conducted to assess this association in adults. We aimed to evaluate the association between dietary fiber intake and allergic diseases (asthma, allergic rhinitis, and atopic dermatitis) among 10,479 adults using data from the Korean National Health and Nutrition Examination Survey (2010–2011). As dietary fiber intake increased, the prevalence of asthma (Q4 adjusted odds ratio (OR): 0.656; 95% confidence interval (CI): 0.48–0.91, *p* for trend < 0.0001) and atopic dermatitis (Q3 crude OR: 0.746; 95% CI: 0.57–0.98; Q4 adjusted OR: 0.712; 95% CI: 0.50–1.01, *p* for trend < 0.0001) decreased. The prevalence of allergic rhinitis (Q2 adjusted OR: 0.840; 95% CI: 0.70–1.00, *p* for trend < 0.0001) tended to decrease, especially in males. Subgroup analysis revealed that fiber intake reduced allergic rhinitis symptoms, including watery rhinorrhea (Q3 adjusted OR: 0.734; 95% CI: 0.55–0.97; Q4 adjusted OR: 0.722; 95% CI: 0.54–0.97) and dog allergen sensitization (Q3 adjusted OR: 0.319; 95% CI: 0.13–0.82; Q4 adjusted OR: 0.338; 95% CI: 0.13–0.86), exclusively in males. Thus, dietary fiber intake influences allergic diseases in adults, especially males.

## 1. Introduction

Dietary fibers can be defined as a group of carbohydrates found in plant-origin foods that are not digested or absorbed by the human body [1]. More specifically, dietary fibers include polysaccharides, oligosaccharides, lignin, and resistant starch [2]. Epidemiological studies have widely reported associations of dietary fiber intake with reduced risks of various diseases, including cardiovascular [3,4], metabolic [5,6], gastrointestinal [7,8], and pulmonary diseases [9] and malignancies [10,11]. Furthermore, increased dietary fiber intake influences the composition of the intestinal microbiota [12], thereby reducing chronic inflammation [13] and improving immune function [14].

Allergy is a damaging immune response by the body against a substance that the body has become hypersensitive toward [15]. As the prevalence of allergic diseases, including asthma, allergic rhinitis, and atopic dermatitis, increases up to 40% in the global population [16], the importance of the association between dietary fiber intake and allergic diseases increases. Many studies evaluated the associations between dietary fiber intake and allergic diseases, including asthma, allergic rhinitis, and atopic disease, especially in the prenatal period and during infancy and childhood [17,18,19]. Prenatal fiber treatment viva maternal dietary fiber intake showed a reduction in the occurrence of infant eczema [20] and protection against infant wheezing [21]. Previous studies have shown no effect of dietary fibers on food allergies among children [22], with some conflicting results regarding eczema, allergic rhinitis, and asthma. Large trials on fiber treatment in children with eczema failed to show beneficial effects [23]; however, most recent studies reported improved symptoms of eczema in the fiber consumption group compared with the placebo group [24]. Several double-blind, placebo-controlled studies showed no beneficial effects in children with asthma and allergic rhinitis in the fiber-treated group [25]; however, a recent study among school-aged children with asthma and allergic rhinitis showed significantly reduced clinical symptoms and improved pulmonary functions with increased dietary fiber intake [26]. However, there is not much information about dietary fiber intake and allergic diseases in adults.

Herein, we assessed the association of dietary fiber intake with allergic diseases, including asthma, allergic rhinitis, and atopic dermatitis, in adults based on data from a large nationwide survey in South Korea.

## 2. Materials and Methods

### 2.1. Study Population

This cross-sectional study obtained data from the Korean National Health and Nutrition Examination Survey (KNHANES) conducted from 2010 to 2011. KNHANES is a nationwide survey that represents the nonindustrialized South Korean population by extracting samples using a stratified multistage-clustered probability sampling design. The survey consisted of a health interview, nutritional survey, and physical examination. The fifth KNHANES, with the participation of the Korean Society of Otorhinolaryngology-Head and Neck, detailed medical interviews, and endoscopic otorhinolaryngological examination, was conducted by residents of the otorhinolaryngology department.

**Figure 1:**
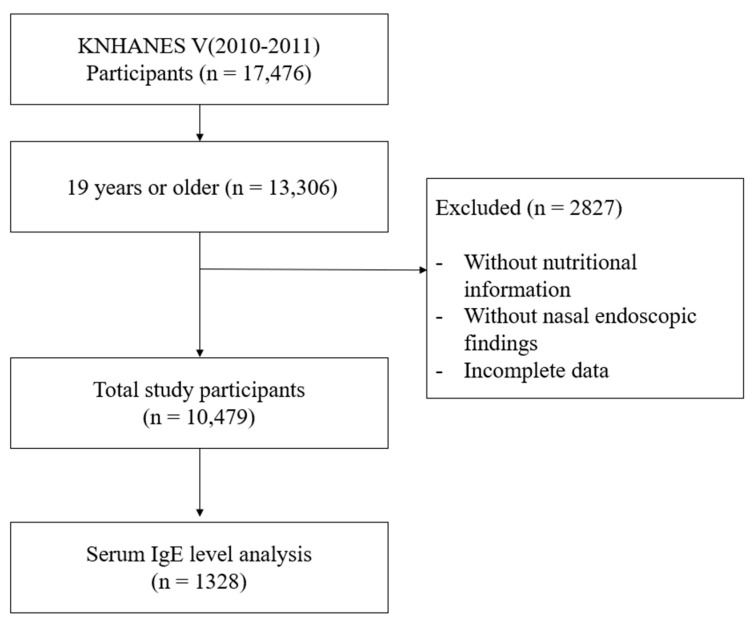
Flow chart for study population selection. KNHANES: Korea National Health and Nutrition Examination Survey.

Among 17,476 participants, those aged under 19 years, without nutritional intake information, without nasal endoscopic examination, or with incomplete data, were excluded. Finally, a total of 10,479 participants were enrolled in this study (Figure 1). The baseline characteristics of participants are shown in Supplementary Table S1.

### 2.2. Diagnosis of Allergic Diseases and Assessment of Related Conditions

Allergic diseases, including asthma, allergic rhinitis, and atopic dermatitis, were defined based on whether the participants had ever been diagnosed with asthma/allergic rhinitis/atopic dermatitis by a physician.

Asthma-related conditions were determined based on the following question: “Have you experienced wheezing or whistling sounds during breathing in the last one year?” and “Have you experienced wheezing or whistling sounds when you breathe while or after exercising in the last one year?” Allergic rhinitis-related conditions were determined based on the question “Have you ever experienced rhinitis symptoms, including rhinorrhea, itching sensation of nose, and sneezing, in the last one year?” and based on nasal endoscopic findings of pale mucosa or watery rhinorrhea before mucosal shrinkage.

In 2010, serum total immunoglobulin E (IgE) and specific IgE levels for three common indoor allergens (house dust mite, dog, and cockroach) were measured in 10% of the total participants via ImmunoCAP. The cut-off values for total IgE and specific IgEs were defined as 100 kU/L and 0.35 kU/L, respectively.

### 2.3. Assessment of Daily Nutritional Intake

Daily intake of nutrients, including energy, carbohydrate, protein, fat, fiber, and water, was investigated based on questionnaires about the participants’ consumption frequency of 63 common Korean foods in the previous year. The daily amount of each nutrient, including dietary fiber, was quantified using the nutrient database provided by the Korea Health and Industry of Development Institute (Ministry of Health and Welfare, 2010). Participants were classified into quartiles (Q1–Q4) according to the amount of dietary fiber intake.

### 2.4. Assessment of Other Variables

Characteristics of participants, including age, sex, household income, residency, alcohol consumption, smoking status, body mass index, and physical activity, were assessed. Household income was categorized into the four following groups with respect to quartiles: <25%, 25–50%, 51–75%, and ≥75%. Residency was classified as urban and rural areas according to the official addresses of the participants. Participants were classified into two groups according to their alcohol consumption ≥1 time a month. Regarding smoking habits, participants were divided into two groups: current smokers and ex-smokers/nonsmokers. Additionally, participants were classified into two groups depending on whether they performed moderate physical activity for more than 20 min more than 5 days a week.

### 2.5. Statistical Analysis

Statistical analysis was conducted using the Statistical Analysis System (SAS) version 9.4 (SAS Institute, Inc., Cary, NC, USA). Univariate and multivariate logistic regression analyses were conducted to evaluate the association between dietary fiber intake and each allergic disease or related condition. Odds ratios (ORs) and 95% confidence intervals (CIs) were calculated. For multivariate logistic regression analysis, confounding factors, including age, sex, residency, household income, smoking status, alcohol consumption, physical activity, BMI, and amount of nutrients (energy, carbohydrate, protein, fat, and water intake), were used. *p* values < 0.05 were considered statistically significant. Furthermore, subgroup analysis according to sex was performed, and the *p*-value for interaction was calculated to investigate the effect of sex on the relationship between dietary fiber and allergy.

## 3. Results

### 3.1. Association between Fiber Intake and Allergic Diseases

Table 1 shows the daily amount of nutritional intake according to the presence of each allergic disease. The allergic rhinitis group had a greater energy intake (1973.80 ± 866.38) than the population without allergic rhinitis (2034.46 ± 829.07) (*p* = 0.013), whereas participants with asthma (1881.07 ± 759.46) had a lesser amount of energy intake compared to participants without asthma (1986.63 ± 866.53) (*p* = 0.003). Participants with either allergic rhinitis or atopic dermatitis showed greater intake of protein, fat, and water compared to those without disease. In contrast, subjects with asthma had a lesser intake of protein, fat, and water than those without asthma. The amount of fiber intake was lesser in participants with either allergic rhinitis (*p* = 0.045), atopic dermatitis (*p* = 0.011) or asthma (*p* = 0.009) compared to the population without disease.

**Figure 2:**
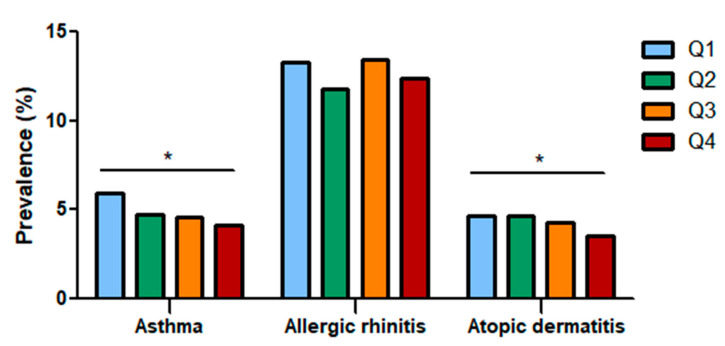
Prevalence of asthma, allergic rhinitis, and atopic dermatitis according to total fiber intake quartiles. The prevalence of asthma and atopic dermatitis decreased as dietary fiber intake increased. Q: Quartile. *: Statistically significant.

The study population included 10,479 participants who were categorized into quartiles based on dietary fiber intake. The prevalence of asthma, allergic rhinitis, and atopic dermatitis in each group was analyzed (Figure 2). As total fiber intake increased, the prevalence of asthma (Q1: 5.90%, Q2: 4.74%, Q3: 4.55%, Q4: 4.15%) and atopic dermatitis (Q1: 4.65%, Q2: 4.66%, Q3: 4.25%, Q4: 3.51%) decreased, showing that total fiber intake influences allergic diseases; however, the prevalence of allergic rhinitis showed no definite association (Q1: 13.25%, Q2: 11.81%, Q3: 13.47%, Q4: 12.39).

Logistic regression analysis revealed that the prevalence of asthma decreased in Q3 (crude OR: 0.761; 95% CI: 0.60–0.97) and Q4 (crude OR: 0.691; 95% CI: 0.54–0.89). The prevalence of atopic dermatitis decreased in Q4 (crude OR: 0.746; 95% CI: 0.57–0.98) (Table 2). After adjusting for confounding factors, the prevalence of asthma decreased (Q4 adjusted OR: 0.656, 95% CI: 0.48–0.91, *p* for trend < 0.0001) and the prevalence of allergic rhinitis (Q2 adjusted OR: 0.840; 95% CI: 0.70–1.00, *p* for trend < 0.0001) and atopic dermatitis (Q4 adjusted OR: 0.712; 95% CI: 0.70–1.01, *p* for trend < 0.0001) tended to decrease as fiber intake increased.

### 3.2. Association between Fiber Intake and Allergic Diseases with Respect to Sex

Since sex might influence the association between fiber intake and allergic diseases, the association between fiber intake and allergic diseases was analyzed according to participants’ sex (Table 3). In males, after adjusting for confounding factors, fiber intake showed significant negative associations with allergic rhinitis (Q2 adjusted OR: 0.704; 95% CI: 0.51–0.97), asthma (Q3 adjusted OR: 0.589; 95% CI: 0.39–0.90; Q4 adjusted OR: 0.523; 95% CI: 0.39–0.90), and atopic dermatitis (Q4 adjusted OR: 0.568; 95% CI: 0.35–0.91). In females, no significant association was identified.

### 3.3. Association between Fiber Intake and Asthma-Related Conditions

The association between fiber intake and asthma-related conditions was analyzed (Table 4). Wheezing sounds while breathing and exercise-induced exacerbation showed no significant relationships with fiber intake in multivariate logistic regression analysis according to the participants’ sex.

### 3.4. Association between Fiber Intake and Allergic Rhinitis-Related Conditions

Multivariate logistic regression analysis of the association between fiber intake and allergic rhinitis symptoms and endoscopic findings showed the tendency for an inverse association between fiber intake and watery rhinorrhea (Table 5). Logistic regression analysis according to participants’ sex showed a significant inverse association in fiber intake and watery rhinorrhea in males (Q3 adjusted OR: 0.734; 95% CI: 0.55–0.97; Q4 adjusted OR: 0.722; 95% CI: 0.54–0.97).

### 3.5. Association between Fiber Intake and Serum IgE Level

The association between fiber intake and serum IgE levels was analyzed (Table 6). No association was found between fiber intake and total IgE levels in both sexes; however, the association between dog allergen-specific IgEs and fiber intake was significant in males (Q3 adjusted OR: 0.319; 95% CI: 0.13–0.82; Q4 adjusted OR: 0.338; 95% CI: 0.13–0.86).

## 4. Discussion

In the past decades, the prevalence of allergic diseases has increased considerably, and it has been suggested that industrialization and lifestyle changes, including dietary habits, could be relevant. This study used a nationwide survey to evaluate the association of allergic diseases and related conditions with dietary fiber intake and analyzed sex differences in their association. In the present study, higher dietary fiber intake was associated with a lower prevalence of allergic rhinitis, asthma, and atopic dermatitis, especially in the male population. Among disease-related conditions, the occurrence of allergic diseases has increased, especially in watery rhinorrhea based on nasal endoscopic examination in the male population, while a wheezing sound had no association with dietary fiber intake. Additionally, among the three indoor allergens investigated, only dog allergens showed associations with fiber consumption.

The prevalence of allergic diseases has increased over the last decades, especially in developed countries. Thus, it is suggested that the shift to western dietary habits with an increased consumption of fat-rich food and a decreased consumption of fruits and vegetables could affect allergic immune responses [27]. In several experimental studies, the importance of a fiber-rich diet has recently been emphasized owing to its contribution to the diversity and function of gastrointestinal microbiota and the production of short-chain fatty acids (SCFAs), which have important roles in recruiting immune cells and regulating inflammatory responses [28]. Previous experimental studies have reported that a high intake of dietary fiber, followed by elevated SCFA levels, could lower airway allergic inflammation by inhibiting inflammatory pathways in dendritic cells and macrophages, inducing regulatory T-cell development, and maintaining epithelial barrier integrity [29,30]. Regarding allergic rhinitis, a dietary fiber intake allergic rhinitis murine model showed less eosinophil infiltration, less goblet cell metaplasia in the nasal mucosa and the lungs, and decreased Th2 cytokines compared with the low-fiber intake model, and the attenuated inflammatory response was accompanied by a significant modulation of the gut microbiota composition [31]. Epidemiologic studies on the association between fiber intake and allergic diseases have focused on the preventive effects of dietary fiber against asthma and atopic dermatitis among children; however, many studies have not been conducted to assess those effects in adults [32]. Recently, a French study of 35,380 participants reported that the highest quantile of total dietary fiber was associated with fewer asthma symptoms and greater disease control in adults [33]. Similarly, in our study, participants with allergic diseases had a lesser intake of dietary fiber despite a higher intake of total energy or other nutrients. Furthermore, the inverse association between fiber intake and the prevalence of allergic diseases in adults was consistent with that reported previously.

In the present study, among various allergic disease-related conditions, only watery rhinorrhea (based on nasal endoscopic examination) had a significant inverse association with dietary fiber intake. Watery rhinorrhea is a representative symptom of allergic rhinitis caused by histamine released from mast cells. Dietary fiber inhibits mast cell activation by reducing calcium entry, attenuating JKN/p38 phosphorylation, and reducing histone deacetylase activity, thus resulting in a decreased release of inflammatory mediators such as histamine [34,35]. It is presumed that the greater effect of fiber intake on watery rhinorrhea compared with that on other factors such as wheezing or nasal congestion could be because of its effect on mast cells. Our investigation of the association between dietary fiber intake and aeroallergen sensitization revealed that higher fiber intake was associated with a lower chance of dog allergen sensitization; however, no association was identified with specific IgEs for other allergens or total IgEs. As mentioned above, previous studies reported that dietary fiber intake contributes to diversity in gastrointestinal microbiota, which eventually regulates inflammatory responses. Although microbial diversity is associated with a lower chance of house dust mite sensitization, no previous studies have evaluated the association between allergen sensitization and fiber intake according to allergen type. Further research is needed on the mechanism of different effects of dietary fiber intake depending on the allergen type [36].

In our study, the association between dietary fiber consumption and allergic diseases was prominent in the male population with respect to several aspects, including the prevalence of diseases, watery rhinorrhea, and dog allergen sensitization. The presence of sex differences in the association between fiber intake and immune or health statuses has been previously reported [37]. Unless the underlying mechanism is not clearly understood, it is suggested that differences in immune functions between sexes as well as sex hormones could affect microbial composition, thus resulting in different dietary effects [38,39]. Furthermore, consumption of fibers from different sources, mainly grains for men and fruits for women, could contribute to this difference, in that different health benefits of cereals, fruits, and vegetables-originated fibers have been reported [40].

The strength of this study is that the data source was a nationwide survey, and the association between dietary fiber intake and allergic disease was analyzed based on detailed interviews and objective methods, including endoscopic examination. This study had several limitations. First, owing to the nature of the cross-sectional design, causal associations could not be assessed. Nevertheless, because endoscopic examination findings were obtained at the time of the survey and responses to the food intake questionnaire were obtained through recalls for the past year, it could be considered that fiber intake affected watery rhinorrhea. Second, we could not investigate atopic dermatitis-related symptoms/signs. Furthermore, there was no objective assessment of asthma because of the lack of detailed questionnaires or physical examinations.

## 5. Conclusions

In conclusion, the study findings suggest that high fiber intake was associated with a decreased prevalence of allergic diseases in adults, especially in the male population. Furthermore, in allergic patients with watery rhinorrhea and dog allergen sensitization, dietary intervention to increase fiber intake could be beneficial for disease control.

## Figures and Tables

**Table 1:** Comparison of daily nutritional intake according to the presence of allergic rhinitis, atopic dermatitis, and asthma with nutrients.

	Allergic Rhinitis			Atopic Dermatitis			Asthma		
	No (N = 9147)	Yes (N = 1334)	*p*-Value	No (N = 10,034)	Yes (N = 447)	*p*-Value	No (N = 9974)	Yes (N = 507)	*p*-Value
Energy (kcal, mean, SD)	1973.80 ± 866.38	2034.46 ± 829.07	0.013	1977.79 ± 856.06	2065.29 ± 981.72	0.065	1986.63 ± 866.53	1881.07 ± 759.46	0.003
Protein (g, mean, SD)	69.98 ± 40.52	75.89 ± 40.20	<0.0001	70.52 ± 40.10	75.34 ± 49.05	0.042	70.98 ± 40.65	65.78 ± 37.68	0.003
Fat (g, mean, SD)	38.05 ± 31.82	45.54 ± 31.96	<0.0001	38.68 ± 31.77	46.28 ± 34.78	<0.0001	39.29 ± 31.87	33.52 ± 32.67	<0.0001
Carbohydrates (g, mean, SD)	322.92 ± 125.00	319.59 ± 122.66	0.363	322.65 ± 124.23	319.05 ± 135.08	0.581	322.54 ± 125.09	321.50 ± 117.02	0.846
Water (g, mean, SD)	990.78 ± 699.08	1057.04 ± 619.57	<0.0001	995.23 ± 686.32	1088.57 ± 758.98	0.010	1005.41 ± 692.06	877.20 ± 632.04	<0.0001
Fiber (g, mean, SD)	7.70 ± 8.13	7.38 ± 4.85	0.045	7.68 ± 7.88	7.01 ± 5.33	0.011	7.69 ± 7.90	7.07 ± 4.99	0.009

**Table 2:** Logistic regression analysis of the association between fiber intake and the prevalence of allergic diseases.

Fiber Intake	Crude Odds Ratio (95% Confidence Interval)			Adjusted Odds Ratio ^a^ (95% Confidence Interval)		
	Allergic Rhinitis	Asthma	Atopic Dermatitis	Allergic Rhinitis	Asthma	Atopic Dermatitis
Q 1	1 (ref)	1 (ref)	1 (ref)	1 (ref)	1 (ref)	1 (ref)
Q 2	0.877 (0.74–1.03)	0.793 (0.62–1.01)	1.004 (0.78–1.30)	0.840 (0.70–1.00)	0.797 (0.62–1.03)	0.997 (0.76–1.31)
Q 3	1.019 (0.87–1.20)	0.761 (0.60–0.97)	0.911 (0.70–1.18)	1.006 (0.84–1.21)	0.777 (0.59–1.02)	0.895 (0.67–1.20)
Q 4	0.925 (0.79–1.09)	0.691 (0.54–0.89)	0.746 (0.57–0.98)	0.948 (0.77–1.17)	0.656 (0.48–0.91)	0.712 (0.50–1.01)
*p* for trend	0.239	0.025	0.120	<0.0001	<0.0001	<0.0001

^a^ Adjusted for age, sex, residency, household income, smoking status, alcohol consumption, physical activity, BMI, energy, carbohydrate, protein, fat, and water intake.

**Table 3:** Multivariate logistic regression analysis of the association between fiber intake and the prevalence of allergic diseases according to sex.

Fiber Intake	Adjusted Odds Ratio ^a^ (95% Confidence Interval)	
	Male	Female
**Allergic rhinitis**		
Q 1	1 (ref)	1 (ref)
Q 2	0.704 (0.51–0.97)	0.902 (0.73–1.11)
Q 3	0.862 (0.64–1.17)	1.081 (0.87–1.34)
Q 4	0.898 (0.66–1.23)	0.935 (0.73–1.20)
**Asthma**		
Q 1	1 (ref)	1 (ref)
Q 2	0.734 (0.49–1.10)	0.807 (0.58–1.12)
Q 3	0.589 (0.39–0.90)	0.918 (0.66–1.29)
Q 4	0.523 (0.34–0.82)	0.756 (0.51–1.12)
**Atopic dermatitis**		
Q 1	1 (ref)	1 (ref)
Q 2	0.695 (0.45–1.09)	1.200 (0.86–1.68)
Q 3	0.653 (0.42–1.02)	1.067 (0.74–1.54)
Q 4	0.568 (0.35–0.91)	0.778 (0.50–1.21)

^a^ Adjusted for age, sex, residency, household income, smoking status, alcohol consumption, physical activity, BMI, energy, carbohydrate, protein, fat, and water intake.

**Table 4:** Multivariate logistic regression analysis of the association between fiber intake and asthma-related conditions.

Fiber Intake	Adjusted Odds Ratio ^a^ (95% CI)	
	Male	Female
	**Wheezing sound during breathing**	
Q 1	1 (ref)	1 (ref)
Q 2	0.806 (0.33–1.95)	0.786 (0.40–1.56)
Q 3	0.641 (0.25–1.65)	0.965 (0.48–1.93)
Q 4	1.341 (0.53–3.39)	1.059 (0.48–2.33)
	**Exercise-induced exacerbation**	
Q 1	1 (ref)	1 (ref)
Q 2	0.779 (0.30–2.02)	0.478 (0.21–1.07)
Q 3	0.906 (0.33–2.47)	0.727 (0.33–1.59)
Q 4	0.516 (0.17–1.53)	0.975 (0.41–2.33)

^a^ Adjusted for age, sex, residency, household income, smoking status, alcohol consumption, physical activity, BMI, energy, carbohydrate, protein, fat, and water intake.

**Table 5:** Multivariate logistic regression analysis of the association between fiber intake and allergic rhinitis-related conditions.

Fiber Intake	Adjusted Odds Ratio ^a^ (95% CI)	
	Male	Female
	**Rhinitis symptoms**	
Q 1	1 (ref)	1 (ref)
Q 2	0.806 (0.33–1.95)	0.786 (0.40–1.56)
Q 3	0.641 (0.25–1.65)	0.965 (0.48–1.93)
Q 4	1.341 (0.53–3.39)	1.059 (0.48–2.33)
	**Endoscopic finding: Pale mucosa**	
Q 1	1 (ref)	1 (ref)
Q 2	0.779 (0.30–2.02)	0.478 (0.21–1.07)
Q 3	0.906 (0.33–2.47)	0.727 (0.33–1.59)
Q 4	0.516 (0.17–1.53)	0.975 (0.41–2.33)
	**Endoscopic finding: Watery rhinorrhea**	
Q 1	1 (ref)	1 (ref)
Q 2	0.903 (0.68–1.19)	0.786 (0.64–0.97)
Q 3	0.734 (0.55–0.97)	0.913 (0.74–1.13)
Q 4	0.722 (0.54–0.97)	0.957 (0.75–1.22)

^a^ Adjusted for age, sex, residency, household income, smoking status, alcohol consumption, physical activity, BMI, energy, carbohydrate, protein, fat, and water intake.

**Table 6:** Logistic regression analysis of the association between fiber intake and serum immunoglobulin E (IgE) levels.

Fiber Intake	Adjusted Odds Ratio ^a^ (95% CI)	
	Male	Female
	**Dog (≥0.35 kU/L)**	
Q 1	1 (ref)	1 (ref)
Q 2	0.600 (0.26–1.41)	1.013 (0.37–2.80)
Q 3	0.319 (0.13–0.82)	0.397 (0.10–1.56)
Q 4	0.338 (0.13–0.86)	0.968 (0.31–3.05)
	**Cockroach (≥0.35 kU/L)**	
Q 1	1 (ref)	1 (ref)
Q 2	1.009 (0.59–1.73)	1.056 (0.60–1.87)
Q 3	0.753 (0.44–1.29)	0.670 (0.35–1.28)
Q 4	0.763 (0.44–1.32)	1.033 (0.53–2.01)
	**House dust mite (≥0.35 kU/L)**	
	1 (ref)	1 (ref)
	1.092 (0.66–1.81)	1.204 (0.81–1.79)
	1.113 (0.68–1.82)	0.939 (0.61–1.44)
	1.157 (0.70–1.90)	1.113 (0.68–1.82)
	**Total IgE (≥100 kU/L)**	
Q 1	1 (ref)	1 (ref)
Q 2	0.874 (0.53–1.45)	1.078 (0.73–1.59)
Q 3	1.145 (0.70–1.88)	0.788 (0.52–1.20)
Q 4	1.070 (0.65–1.77)	1.064 (0.66–1.71)

^a^ Adjusted for age, sex, residency, household income, smoking status, alcohol consumption, physical activity, BMI, energy, carbohydrate, protein, fat, and water intake.

## Supplementary Materials

The following are available online at https://www.mdpi.com/1660-4601/18/6/2889/s1, Table S1. Baseline characteristics of participants according to the presence of allergic rhinitis, atopic dermatitis and asthma.
