# Supplementary material for: Association of Allergic Diseases and Related Conditions with Dietary Fiber Intake in Korean Adults
Source: Int J Environ Res Public Health. 2021 Mar 12;18(6):2889. doi: 10.3390/ijerph18062889 (PMC7998737; doi:10.3390/ijerph18062889)
Supplement: Supplementary file 1 [file ijerph-18-02889-s001.pdf]

International Journal of
*Environmental Research and Public Health*

MDPI

**Table S1.** Baseline characteristics of participants according to the presence of allergic rhinitis, atopic dermatitis and asthma.

| Variables | Allergic rhinitis | | | Atopic dermatitis | | | Asthma | | |
|---|---|---|---|---|---|---|---|---|---|
| | No (N[a]=9,147) | Yes (N[a]=1,334) | P-value | No (N[a]=10,034) | Yes (N[a]=447) | P-value | No (N[a]=9,974) | Yes (N[a]=507) | P-value |
| Age | 52.18 ± 16.17[b] | 42.68 ± 14.39[b] | <0.001 | 51.38 ± 16.16[b] | 41.81 ± 15.94[b] | <0.001 | 50.62 ± 16.15[b] | 57.98 ± 17.10[b] | <0.001 |
| Sex | | | 0.003 | | | 0.386 | | | 0.496 |
| Male | 3778 (41.3) | 494 (37.03) | | 4081(40.67) | 191 (42.73) | | 4058 (40.69) | 214 (42.21) | |
| Female | 5369 (58.7) | 840 (62.97) | | 5953 (59.33) | 256 (57.27) | | 5916 (59.31) | 293 (57.79) | |
| Residency | | | <0.001 | | | <0.001 | | | 0.304 |
| Urban | 7015 (76.69) | 1158 (86.81) | | 7794 (77.68) | 379 (84.79) | | 7787 (78.07) | 386 (76.13) | |
| Rural | 2132 (23.31) | 176 (13.19) | | 2240 (22.32) | 68 (15.21) | | 2187 (21.93) | 121 (23.87) | |
| Household income | | | <0.001 | | | 0.004 | | | <0.001 |
| <25% | 2038 (22.48) | 142 (10.73) | | 2116 (21.28) | 64 (14.41) | | 1987 (20.10) | 193 (38.29) | |
| 25–50% | 2316 (25.55) | 337 (25.47) | | 2539 (25.53) | 114 (25.68) | | 2535 (25.65) | 118 (23.41) | |
| 50–75% | 2400 (26.48) | 451 (34.09) | | 2715 (27.30) | 136 (30.63) | | 2745 (27.77) | 106 (21.03) | |
| >75% | 2311 (25.49) | 393 (29.71) | | 2574 (25.88) | 130 (29.28) | | 2617 (26.48) | 87 (17.26) | |
| Smoking | | | 0.003 | | | <0.001 | | | 0.822 |
| Current smoker | 7376 (80.76) | 1121 (84.22) | | 8164 (81.5) | 333 (74.5) | | 8085 (81.18) | 412 (81.58) | |
| Ex/non-smoker | 1757 (19.24) | 210 (15.78) | | 1853 (18.5) | 114 (25.5) | | 1874 (18.82) | 93 (18.42) | |
| Alcohol consumption | | | <0.001 | | | 0.015 | | | 0.026 |
| ≥1 time a month | 4476 (49.27) | 574 (43.52) | | 4861 (48.8) | 189 (42.86) | | 4781 (48.30) | 269 (53.37) | |
| <1 time a month | 4608 (50.73) | 745 (56.48) | | 5101 (51.2) | 252 (57.14) | | 5118 (51.70) | 235 (46.63) | |
| Physical activity | | | 0.7294 | | | | | | 0.707 |
| >5days/week | 8235 (90.23) | 1196 (89.92) | | 9032 (90.23) | 399 (89.26) | | 8978 (90.21) | 453 (89.70) | |
| ≤5days/week | 892 (9.77) | 134 (10.08) | | 978 (9.77) | 48 (10.74) | | 974 (9.79) | 52 (10.30) | |

| | | | | | | | | | |
|---|---|---|---|---|---|---|---|---|---|
| BMI | 23.67 | 23.2 | <0.001 | 23.61 | 23.55 | 0.7459 | 23.59 | 24.02 | 0.0056 |

[a] number of study population; [b], mean age ± standard deviation; brackets, percentage of each group population.
